# Persistent Retinal Detachment in Retinoblastoma: The Challenges

**DOI:** 10.1155/2020/1486757

**Published:** 2020-09-10

**Authors:** Sophia El Hamichi, Dhariana Acon, Veronica Kon Graversen, Aaron S. Gold, Audina M. Berrocal, Timothy G. Murray

**Affiliations:** ^1^Miami Ocular Oncology and Retina, Miami, FL, USA; ^2^Department of Ophthalmology, Bascom Palmer Eye Institute, University of Miami Miller School of Medicine, Miami, FL, USA

## Abstract

**Introduction:**

Retinoblastoma (RB) is the most common eye tumor in children. There have been significant improvements in treatment options targeting killing the tumor while also conserving the eye and attempting to conserve functional vision. Retinal detachment (RD) is not an uncommon event and compromises the vision and sometimes RB treatment.

**Materials and Methods:**

Retrospective review of 62 patients over a period of 8 years between 2012 and 2019 with eyes treated for RB and having persistent RD that did not resolve after complete tumor regression.

**Results:**

Forty-two patients of these 62 cases developed RD (67%). The RD resolved in 35 patients (83% of RD), and 7 patients (16% of RD) developed a persistent RD. In all the persistent RD groups (7 patients/11 eyes), RB and RD were present simultaneously in the first ophthalmological assessment. Sex ratio was 2 females/5 males. The mean age of diagnosis was 11 months. All eyes had advanced RB stages. Eight eyes had local treatment with transpupillary laser, 6 eyes received IAC, and 3 patients received systemic chemotherapy. In 9 eyes, the RD had both exudative and tractional components. Only one eye had a pure tractional RD due to persistent fetal vasculature, and one eye had rhegmatogenous RD component with presence of a tear in addition to exudation. None of the eyes received RD surgical repair.

**Conclusion:**

Persistent RD occurs in eyes with advanced RB stages with complex RD with more than one component. The dilemma is performing a vitrectomy in eyes with cancer and poor visual outcome.

## 1. Introduction

The first description of retinoblastoma (RB) was made by Pawius in the 16^th^ century. The tumor was originally referred to as fungus hematodes, and enucleation was the treatment of choice [1]. Retinal detachment (RD) is commonly associated with RB and can be seen as part of the initial presentation or as a result of treatment in these patients. Nevertheless, RD is not a criterion in any of the classification systems known for RB, and most of the patients presenting with RD have traditionally been enucleated [1].

During the last two decades, more than 400 years later, a dramatic change in the management of RB has taken place. Currently, most RBs, including advanced stages, are being managed by globe conserving treatments [1]. A globe conserving approach has the advantage of sparing these young children the negative psychological impact enucleation can cause, although removal of the eye is unavoidable in some cases. Furthermore, the aspiration of conserving any functional vision in these young patients is increasingly put into consideration, as a better visual function helps ensure a better quality of life.

The challenges presented by persistent RD include poor visual outcome, risk of progression to neovascular glaucoma with painful eye, phthisis bulbi, and in some cases difficulty to access the tumor during retinoblastoma treatment.

In this study, the authors review and describe persistent RD in eyes with RB, its etiologies, the treatment attempts, and the end result in this subgroup of patients.

## 2. Materials and Methods

We conducted a retrospective study of eyes of patients diagnosed with retinoblastoma, treated between June 2012 and December 2019, within the Miami Ocular Oncology and Retina (MOOR), Miami, Florida, USA. We reviewed the clinical records of 62 patients; from there, charts of patients with persistent RD after complete treatment and tumor regression were selected. Data collected include the age of the patient, gender, age at RB diagnosis, family history, laterality of RB, RB stage (according to Reese-Ellsworth classification), treatment received for RB, age at RD, laterality and type of RD, and evolution of the eye.

Persistence of RD was defined as the presence of subretinal fluid after completing RB treatment, with total tumor regression.

The study was approved by the institutional Ethics Committee, and data accumulation was carried out in adherence to the tenets of the Declaration of Helsinki.

### 2.1. Patient Eligibility and Exclusion Criteria

We included all the patients with persistent subretinal fluid after completing their cancer treatment with total tumor regression. We did not consider classification of the tumor or type of treatment as an exclusion criterion.

We did not include RB patients with focal or total RD that resolved spontaneously or after scleral repair surgery.

### 2.2. Examination and Treatment Procedures

Patients were examined under general anesthesia for ophthalmological evaluation. All patients underwent complete ophthalmological examination, fundus photographs, A-scan and B-scan ultrasonography, fluorescein angiography, and MRI every six months. Repeated examinations were performed depending on evolution of each case.

Available treatment modalities were laser photocoagulation, cryotherapy, intra-arterial chemotherapy (IAC), intravitreal chemotherapy, systemic chemotherapy, periocular injections of carboplatin as consolidating treatment, external beam radiation therapy (EBRT), and enucleation. Treatment choices were performed by the treating physician after discussion with parents or legal guardians.

## 3. Results

**Figure 1:**
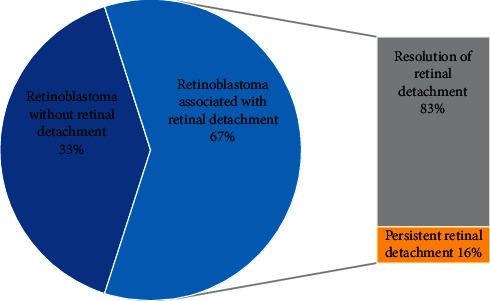
Pie chart illustrating the distribution of retinal detachment during retinoblastoma and its evolution.

In this study, 62 patients were treated for RB over a period of 8 years. Forty-two patients developed RD, which represents 67% of RB patients. Thirty-five patients had a complete resolution of the RD, which represents 56% of total RB patients and 83% of RB associated with RD. Seven patients (10 eyes) experienced a persistent RD, which represents 11% of total patients and 16% of RB associated with RD (Figure 1).

The mean age of diagnosis for both RB and RD was 11 months with the latest diagnosed at 24 months and the earliest at 2 days old because of family history of RB. In all our patients, both RB and RD were present simultaneously and diagnosed during the first ophthalmological assessment (Table 1).

Sex ratio was 2 females/5 males. RB was bilateral in 3 cases and unilateral in 4. The RB stage was 5B in 8 eyes (6 patients) and stage 4 in 2 eyes (1 patient). RD occurred in 10 eyes and was diagnosed at the same time as the RB. Eight eyes (6 patients) had local treatment with transpupillary laser, 6 eyes (5 patients) received IAC, and 3 patients have received systemic chemotherapy including one patient that received both IAC in both eyes and systemic chemotherapy. Only one patient received EBRT and periocular injection of carboplatin as consolidating treatment. None of these patients received cryotherapy or intravitreal chemotherapy injections (Tables 1–3).

**Figure 2:**
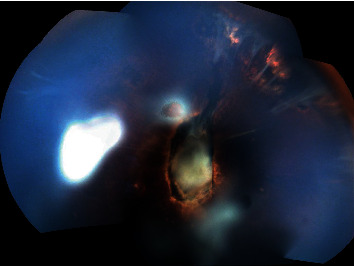
Patient 2 with treated retinoblastoma and persistent tractional retinal detachment associated with persistent fetal vasculature in the right eye.

**Figure 3:**
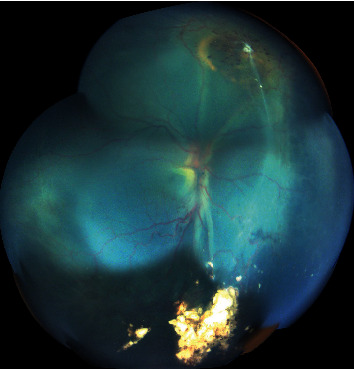
Patient 5 with treated retinoblastoma and persistent complex retinal detachment (tractional and exudative) of the right eye.

In 9 eyes, the RD had exudative and tractional components (Figure 2). One eye had a pure tractional RD due to persistent fetal vasculature (Figure 3). Only one eye had rhegmatogenous RD component with presence of a tear in addition to exudation and traction (Table 2). All eyes had complex RD. None of the eyes received RD surgical repair. The evolution was marked by globe phthisis in one patient's eye and globe prephthisis in another patient's eye (Table 3). Both patients had bilateral RB and bilateral RD with very poor visual outcome. Enucleation was not performed in any of the cases.

## 4. Discussion

### 4.1. Types of Retinal Detachments in Retinoblastoma

Exudative RD, rhegmatogenous RD, and tractional RD may occur in RB.

Exudative RD tends to occur when exophytic RB grows subretinally and is usually associated with subretinal tumor seeding [2]. Typically, as a response to systemic chemotherapy, when the tumor shrinks, the exudative RD resolves spontaneously [2].

Even though systemic chemotherapy is believed to help resolve an exudative RD, in one study, it might have caused the appearance of an exudative RD right after the first cycle of systemic chemotherapy. This was thought to be the result of excessive initial inflammation from the chemoreduction or of rapid shrinkage of the tumor [3].

Also, IAC has been shown to resolve 43% of total RD and 100% of partial RD related RB [4]. On the other hand, cases of exudative RD have been described after IAC, and the RD did not resolve despite the regression of the tumor [3]. One of those cases was a mixed tractional exudative RD after IAC [5].

Rhegmatogenous RD in RB is thought to be due to the focal retinal necrosis and retinal breaks. These retinal breaks and necrosis are secondary to cryotherapy application in the area of the tumor. Additionally, the surrounding area of the tumor usually has an exudative RD already present. Another theory is that cryotherapy increases the focal inflammation in eyes with ongoing inflammation due to the tumor. This may result in traction causing breaks in weakened retinal zones. Furthermore, most of these RB patients undergo chemotherapy, which impairs their wound healing process, making them more susceptible to this complication [6].

There were also cases of rhegmatogenous RD reported after intra-arterial chemotherapy. It is thought to be a direct complication of IAC, and it is explained by the rapid regression of the tumor leaving an atrophic retinal hole or break. Rhegmatogenous RD occurs mostly in advanced stages of RB, with extensive endophytic tumor [7, 8].

Tractional detachment may be related to RB's response to treatment. The latter is associated with vitreoretinal complications including tractional RD, vitreous traction bands, preretinal fibrosis, subretinal fibrosis, and pseudovitreous seeding [9].

Most of the cases described in this study are of complex RD composed of more than one mechanism of RD: 9 eyes had mixed exudative and tractional RD, 1 eye had a combination of rhegmatogenous and tractional RD, and only one eye had solely tractional RD due to persistent fetal vasculature.

### 4.2. Surgery of Retinal Detachment in Retinoblastoma

Operating on RD in eyes harbouring active RB carries high risk of tumor dissemination. There are studies where surgery in RB with rhegmatogenous RD was performed. The surgery consisted in scleral buckling with or without fluid drainage. The risk of tumor dissemination is lower when no fluid drainage is performed [8–14]. Furthermore, redetachment occurred in some of these cases due to the tumor reoccurrence and led to enucleation.

In our study, only one eye presented with rhegmatogenous RD and was associated with significant exudation and traction. In this particular case, scleral buckle alone would have not been efficient due to the extent of detachment.

In our study, RD occurred in 67% of total RB cases. Most of these RD cases which represent 83% of RD associated with RB resolved after RB treatment. Those that persisted represent 16% of all RD associated with RB. These persistent RD cases were complex with more than one mechanism involved. Additionally, in all these cases, RD was already present at RB diagnosis during the first ophthalmological evaluation visit.

Performing pars plana vitrectomy may be the surgical option to attempt restoring the retinal anatomy and potential vision. However, pars plana vitrectomy has a high risk of spreading the tumor and causing metastasis. The main concern for RB patients remains to essentially control the tumor and save the life. Vitrectomy should not be performed until at least 18 months after the patient has received their last treatment session [15]. Nonetheless, this waiting period will significantly affect the result of the surgery and even question its necessity. The decision on operating in these complex cases varies from case to case, and it involves a discussion with parents or legal guardian weighing risks versus benefits. In our study, we did not perform pars plana vitrectomy in any patient, as the risks outweighed the benefits.

### 4.3. Persistent RD: The Outcome

The challenges that are encountered when RD persists during RB are multiple. In some cases, the RD hinders the access to the tumor for local treatment. This can lead to uncontrolled RB with risk of life-threatening dissemination. The approach in this particular scenario is to proceed with enucleation to save the life. In our study, with a strict treatment regimen and close follow-up, we were able to avoid enucleation in all these cases.

Other encountered complications are those of longstanding RD, including loss of vision, neovascular glaucoma with a risk of painful blind eye, and phthisis bulbi [16, 17]. These complications may require enucleation. Furthermore, they have a considerable psychological impact in these children and affect their quality of life. In our study, 1 eye in 1 patient evolved into phthisis bulbi and 1 eye was prephthisical in another patient.

## 5. Conclusions

In this unique study, early retinal detachment associated with advanced retinoblastoma is common. For the majority of eyes, the primary treatment for the retinoblastoma leads to resolution of the exudative retinal detachment in the vast majority of patients (35/42, 83%). Nonresolving retinal detachment requires a focus on etiology, with exudative, tractional, and rhegmatogenous detachments almost always presenting with a combined presentation (often tractional and exudative). In the setting of persistent retinal detachment, we currently recommend observation until complete tumor stability is maintained. At this point, many eyes may have limited visual potential and may benefit from long-term observation. If the retinal detachment is amenable to repair, noninvasive procedures are considered first, but if tumor involution is assured, primary repair utilizing microincisional vitrectomy is most likely to obtain retinal reattachment, often requiring silicone oil tamponade. These eyes extend the complexity of retinoblastoma management beyond primary tumor care and require a broad understanding, and discussion, of relative risks and benefits. Surgical management demands a retina surgical specialist comfortable with intraocular malignancies, pediatric retinal detachments, and unique aspects of surgical repair in this rare cohort. Finally, as with all intraocular malignancies, the focus remains to save the child's life, retain an anatomically stable globe, and recover best visual function.

## Figures and Tables

**Table 1:** Age of retinoblastoma and retinal detachment diagnosis.

Patient	Current age	Gender	Age at retinoblastoma diagnosis	Retinoblastoma laterality	Age at retinal detachment diagnosis	Retinal detachment laterality
**1**	4 yo	Male	7 months	Unilateral	7 months	Unilateral
**2**	3 yo	Male	22 months	Unilateral	22 months	Unilateral
**3**	2 yo	Female	2 days	Bilateral	2 days	Bilateral
**4**	8 yo	Female	12 months	Bilateral	12 months	Bilateral
**5**	8 yo	Male	24 months	Unilateral	24 months	Unilateral
**6**	3 yo	Male	6 months	Unilateral	6 months	Unilateral
**7**	8 yo	Male	6 months	Bilateral	6 months	Unilateral

**Table 2:** Stage of retinoblastoma and type of associated retinal detachment.

Patient	Stage of retinoblastoma (Reese-Ellsworth classification)	Type of retinal detachment
**1**	Stage 5B	Exudative and tractional
**2**	Stage 5B	Tractional with persistent fetal vasculature
**3**	Stage 4	Exudative and tractional
**4**	Stage 5B	Exudative and tractional
**5**	Stage 5B	Rhegmatogenous, exudative, and tractional
**6**	Stage 5B	Exudative and tractional
**7**	Stage 5B	Exudative and tractional

**Table 3:** Treatments received and evolution.

Patient	Transpupillary thermal laser	Cryotherapy	Intra-arterial chemotherapy	Systemic chemotherapy	Additional treatment	Evolution
**1**	Yes	No	Yes	No	None	Stable
**2**	Yes	No	Yes	No	None	Stable
**3**	Yes	No	No	Yes	None	Stable
**4**	No	No	No	Yes	External beam radiotherapy + periocular carboplatin	Phthisical globe OS
**5**	Yes	No	Yes	No	None	Stable
**6**	Yes	No	Yes	Yes	None	Stable
**7**	Yes	No	Yes	Yes	None	Phthisical globe OS

## Data Availability

Relevant raw data from this study are available from the corresponding author upon request.
